# The immunosignature of canine lymphoma: characterization and diagnostic application

**DOI:** 10.1186/1471-2407-14-657

**Published:** 2014-09-08

**Authors:** Stephen Albert Johnston, Douglas H Thamm, Joseph Barten Legutki

**Affiliations:** Flint Animal Cancer Center, Colorado State University, 300 West Drake Road, Fort Collins, CO 80523-1620 USA; Center for Innovations in Medicine, The Biodesign Institute, Arizona State University, Tempe, AZ 85287-5901 USA

**Keywords:** Cancer, Dog, Diagnostic, Antibody response, Peptide microarray

## Abstract

**Background:**

Cancer diagnosis in both dogs and humans is complicated by the lack of a non-invasive diagnostic test. To meet this clinical need, we apply the recently developed immunosignature assay to spontaneous canine lymphoma as clinical proof-of-concept. Here we evaluate the immunosignature as a diagnostic for spontaneous canine lymphoma at both at initial diagnosis and evaluating the disease free interval following treatment.

**Methods:**

Sera from dogs with confirmed lymphoma (B cell n = 38, T cell n = 11) and clinically normal dogs (n = 39) were analyzed. Serum antibody responses were characterized by analyzing the binding pattern, or immunosignature, of serum antibodies on a non-natural sequence peptide microarray. Peptides were selected and tested for the ability to distinguish healthy dogs from those with lymphoma and to distinguish lymphoma subtypes based on immunophenotype. The immunosignature of dogs with lymphoma were evaluated for individual signatures. Changes in the immunosignatures were evaluated following treatment and eventual relapse.

**Results:**

Despite being a clonal disease, both an individual immunosignature and a generalized lymphoma immunosignature were observed in each dog. The general lymphoma immunosignature identified in the initial set of dogs (n = 32) was able to predict disease status in an independent set of dogs (n = 42, 97% accuracy). A separate immunosignature was able to distinguish the lymphoma based on immunophenotype (n = 25, 88% accuracy). The individual immunosignature was capable of confirming remission three months following diagnosis. Immunosignature at diagnosis was able to predict which dogs with B cell lymphoma would relapse in less than 120 days (n = 33, 97% accuracy).

**Conclusion:**

We conclude that the immunosignature can serve as a multilevel diagnostic for canine, and potentially human, lymphoma.

**Electronic supplementary material:**

The online version of this article (doi:10.1186/1471-2407-14-657) contains supplementary material, which is available to authorized users.

## Background

Clinical diagnosis of cancer is a complex process usually initiated by presentation of indicative symptoms. Suspected conditions are identified as possible differential diagnosis and a battery of blood tests, urinalysis, imaging tests, and biopsy are conducted before final diagnosis is made. Biomarkers have been identified for some cancers, but have limited use as a primary screening tool. A single blood test capable of diagnosing cancer with high sensitivity and specificity would enhance patient care by streamlining the diagnostic process. Non-Hodgkin lymphoma (NHL) is a spontaneously occurring neoplasm of particular interest. NHL newly affects approximately 70,000 people annually in the United States [1] and has had a steadily increasing incidence in the United States and Europe [2]. If diagnosed early, effective treatments can be selected [2, 3] and the 5 year survival is 72% [1]. However, diagnosis is complicated by the lack of a non-invasive test and is presently made by clinical signs, physical examination findings and imaging, with confirmation of disease by biopsy. Even with effective treatment, 50% of patients with aggressive lymphomas have residual disease and eventually relapse [4]. A serological test for monitoring lymphoma would have utility at multiple stages: early detection, diagnosis and monitoring of residual disease. Spontaneous canine lymphoma (LSA) and human NHL have nearly identical presentations and pathologies [5–7], making them ideal partner species in which to explore blood based diagnostics. Dogs have been used as predictive models for human oncology in multiple cancers [8], including lymphoma [9, 10]. Here we explore the application of the immunosignature diagnostic to canine LSA.

Lymphoma is one of the most commonly encountered canine neoplasms, generally affecting middle-aged to older dogs. Breeds reported to be at increased risk include boxers, bull mastiffs, Bassett hounds, Saint Bernards, Scottish terriers, Airedales, golden retrievers and English bulldogs [11]. Typically dogs present with an aggressive high-grade multicenteric lymphoma, of which diffuse large B-cell lymphoma (DLBCL) is the most common subtype [5]. Following chemotherapy, 95% all dogs relapse following a period of remission. While approximately 85% of dogs present with multicentric peripheral lymphadenopathy, a small percentage present with visceral disease only (e.g. primary mediastinal, gastrointestinal or hepatosplenic forms), which requires serial imaging in order to monitor remission status. In humans, remission status is monitored by CT, MRI or PET scans [2]. Facile and early detection of relapse may facilitate re-induction of remission and improve outcome. Here, we evaluate the immunosignature diagnostic technology relative to these diagnostic requirements.

A serological test would facilitate routine monitoring during an annual wellness examination, enable faster diagnosis when LSA is suspected and allow monitoring following treatment. Design of such a test is dependent on the identification of an appropriate biomarker. Ideally, this test would be applicable to early disease, but to do so it must overcome the "blood dilution" problem: that is, if 10^6^ initiating cancer cells release 1000 molecules each of a biomarker into two liters of blood at steady state, the concentration of this biomarker would only be 1.3 × 10^-14^ M [12], placing it below the detection limits of even the best assays [12]. Antibodies are an ideal solution to this problem. Self-reactive antibodies have been reported in cancer and autoimmune disease [13, 14]. Arising early in the course of a disease, the activation of a single B cell results in an ~10^11^ amplification of signal in only a week [15]. Furthermore, antibodies are stable in blood, enabling archived samples to be used in assay development or serial monitoring [16, 17].

We have developed a technology termed immunosignatures which displays the circulating antibody repertoire upon an addressable, machine readable random peptide microarray (reviewed in [18]). The random sequences allow an unbiased display of all types of antibody binding. The peptides on the microarray serve as mimetics of the actual epitopes and capitalize on the cross-reactivity of antibodies. Even if the actual epitope is not present, another peptide that the same antibody can bind will be present. In addition, the arrays are inexpensive and can be adapted to high throughput sample processing. Thus far we have been able to distinguish over thirty diseases from healthy individuals with high accuracy and specificity. Antibodies are detected earlier by the immunosignature than an ELISA in infectious disease [18], an immunosignature of Alzheimer’s disease in mouse models is evident months before symptoms begin [19], and vaccine efficacy can be predicted using immunosignatures [20]. The immunosignature is capable of distinguishing types of brain tumor pathologies and molecular subtypes which would otherwise only be diagnosable by biopsy [21]. Each of these distinctions was made using the same immunosignature microarray using species-specific detection reagents. The characteristics of immunosignature diagnostics have been reviewed [18].

In this study we assess the ability of the immunosignature to characterize the humoral response to canine LSA and investigate its clinical utility in diagnosing different subtypes of disease. Pretreatment serum samples from patients presenting with T cell and B cell LSA (LSA-T and LSA-B) are compared to healthy dogs. Serial serum samples from patients that experienced remission following chemotherapy and ultimately relapsed were investigated. Immunosignatures informative for each subtype of disease and their diagnostic efficacy are reported.

## Methods

### Study plan

The diagnosis and treatment of many cancers, including canine and human LSA, is complicated by the lack of a non-invasive serological test. Having demonstrated that the immunosignature is capable of simultaneously classifying human cancers including multiple subtypes of brain cancer [21], we hypothesized that the immunosignature could be applicable to canine LSA. The Colorado State University tumor archive was canvassed to select sera from 38 B cell LSA, 11 T cell LSA and 39 clinically healthy dogs collected as part of ongoing prospective archiving efforts. Summary statistics of age, breed and clinical presentation are described in Table 1.

**Table 1:** **Summary of study population signalment** ^**1**^

Class	N	Age ^2^	Sex ^3^	Breed
Healthy	39	6 (2 to 15)	M 24	Mixed Breed (19), Golden Retriever (6), Labrador Retriever (3), Staffordshire Terrier (2), Australian Cattle Dog (2), Australian Shepherd, Dalmatian, Doberman, German Wire Haired Pointer, Std. Poodle, St. Bernard, Rottweiler
			F 15	
LSA-B	38	7.9 (2 to 13)	M 22	Mixed Breed (10), Golden Retriever (5), Border Collie (4), German Shepard (2), Rottweiler (2), Scottish Terrier (2), Vizsla (2), Bassett Hound, Belgian Malinois, Boxer, Chesapeake Bay Retriever, Collie, Doberman, Labrador Retriever, Miniature Schnauzer, Sheltie, Staffordshire Terrier, Other
			F 16	
LSA-T	11	6.97 (4 to12)	M 4	Golden Retriever (3), Boxer (3), Mixed Breed (2), Bull Mastiff, Irish Setter, Labrador Retriever
			F 7	

^1^Archived serum samples from client owned dogs presenting to the Animal Cancer Center at Colorado State University were used.

^2^Median age is presented with the range, low to high, in parenthesis.

^3^Neutered and intact dogs are totaled under the appropriate sex.

### Patient sera

Serum samples were obtained from clinically normal client-owned dogs or dogs with histologically or cytologically confirmed LSA and stored at -80°C from the time of presentation, prior to any specific therapy, and were collected with owner consent and approval of the CSU Institutional Animal Care and Use Committee (Protocol #10-2007A). Samples were collected during routine visits under nominal clinical conditions. In a subset of patients, sera were collected serially from dogs with LSA at each subsequent recheck visit, up to and including the time of relapse.

### Peptide microarrays

The CIM10Kv2 random peptide microarrays used for immunosignatures have been described previously [17, 19]. These microarrays contain 10,000 random peptides containing 17 random residues and an N-terminal CSG linker. Known peptide sequences were piezo-electrically printed in an addressable format with two printings of the 10,000 peptides per standard slide. Arrays were obtained from the Peptide Array Core at Arizona State University ( http://www.peptidearraycore.com). Two print runs having a quality control technical cross batch correlation of 0.67 were used for this study.

### Binding sera to the immunosignature arrays

Patient serum was used to probe the CIM10Kv2 immunosignaturing microarray as described previously using a Tecan HS4800 [17, 21]. Prior to the assay, unbound peptide was removed by prewashing the arrays in 7.33% acetonitrile, 30% isopropanol and 0.5% trifluoracetic acid. The arrays were then blocked in phosphate buffered saline with 0.05% Tween 20 (PBST), 3% Bovine Serum Albumin (BSA) and 0.014% mercaptohexanol for 1 hour. Following washing with PBST, serum was diluted to 1:500 in incubation buffer (PBST with 3% BSA) for 1 hour at 37°C. Bound IgG was then detected using 5.0 nM anti-dog IgG (Fc gamma specific)-Dylight 649 for 1 hour. Anti-Dog IgG (gamma) from KPL was used in the first batch of arrays and Anti-Dog IgG(gamma) from Jackson Immuno Research was used in the second batch due to discontinuation of the KPL conjugate. The microarrays were then washed in PBST then distilled water. Nitrogen dried slides were then scanned at 633 nm using an Agilent ‘C’ type scanner at 100% laser power and 100% PMT.

### Statistical analysis

Raw array images were aligned using GenePix (Molecular Devices) to produce a tab deliminated results file. Physical artifacts were removed by flagging the features as bad. Results files were evaluated in GeneSpring (Agilent) or Bioconductor R (3.0.1). For all analysis, the arrays run with the KPL and the Jackson Immuno Research conjugates were treated separately. The ComBat algorithm was used to minimize assay batch effects on per chip median normalized scores [22]. Background subtraction based on empty features was applied to all replicates in a comparison as needed. Criteria for selecting informative peptides between classes were a Student’s T-test p value less than 0.05 with the Benjamani and Hochberg False Discovery Rate (FDR) correction and a minimum fold change of 1.5× between class averages. A support vector machine in R (e1071 library) [23] was used for classification with the following settings type = C, Kernel = polynomial, degree = 2, gamma = 0.1, coef0 = 1, epsilon = 0.1 and cost = 1. Iterative testing was done in R by splitting the patient population into 85% training for peptide selection and the remaining 15% into test sets to evaluate classification based on the selected peptides, a minimum fold change of 10 between classes was used for the iterative testing. Heatmaps were generated in GeneSpring with individuals and peptides clustered using the default Pearson correlation settings. Principal component values were obtained in GeneSpring and plotted in GraphPad Prism. Power analysis conducted in R at 80% power, 5.0×10^-6^ significance level (FDR adjusted p value), Standard deviation of 50%, and a 1.5 fold change (delta) between groups indicated a minimum of 11 samples were needed for each comparison made. All comparisons were adequately powered.

## Results

### The immunosignature distinguishes canine lymphoma patients from healthy dogs

**Figure 1:**
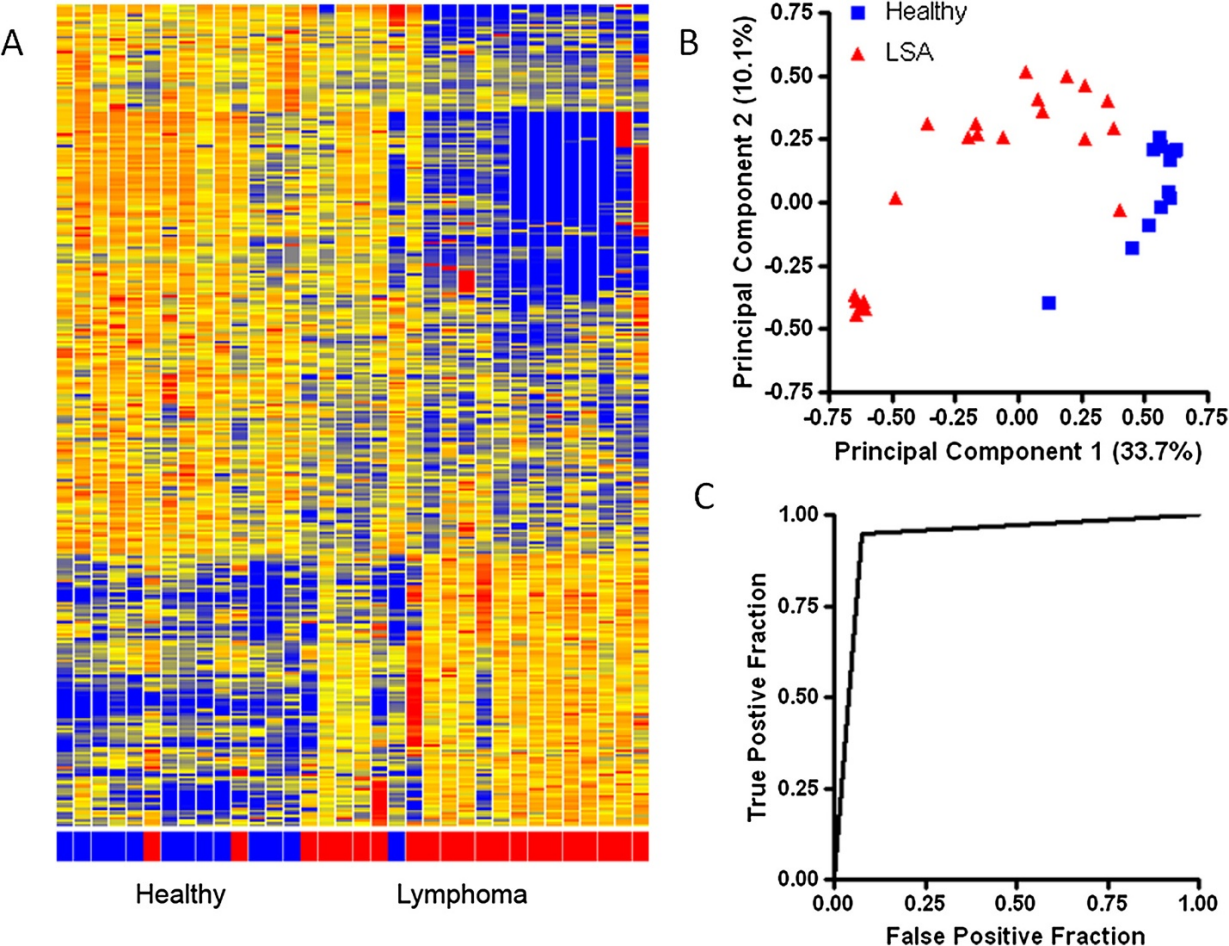
**The immunosignature distinguishes canine lymphoma patients from healthy dogs.** A Student’s T-test (p < 0.05 with FDR) and a 1.5 fold change between classes was used to select 340 informative peptides. The distribution of intensities is shown in the Heatmap **(A)**. Colors represent the per peptide median normalized intensities. Yellow indicates the median, red five-fold above the median and blue 0.25 fold below the median. Each row represents a peptide and each column represents and individual. Individuals were clustered in GeneSpring using the Pearson correlation to each other. Variation among individuals based on the 340 peptides is shown in the PCA plot **(B)** where the first two principal components are plotted. The classification efficacy is plotted in the ROC curve in **(C)**. Print run one and the KPL conjugate were used for this assay.

Initially, the immunosignature was evaluated with a small set of dogs to determine the ability to distinguish LSA (either B or T cell) from healthy. Patient (n = 21) and healthy dog (n = 11) sera were randomized and applied to the CIM10Kv2 array. Per chip median normalized values were ComBat normalized to remove batch effects and then compared between LSA and healthy dogs. A Student’s T-test selected 340 peptides having an FDR corrected p value less than 0.05 and a minimum 1.5 fold difference in intensity between classes. Reactivity is shown in the heatmap in Figure 1A. Separation of healthy and LSA is shown in the principal components analysis (PCA) in Figure 1B. Leave one out cross validation (LOOCV) was able to separate LSA and healthy with 94% accuracy. A receiver-operator characteristic (ROC) curve is shown in Figure 1C. A similar distinction was made using the CIM10Kv1 array, which is comprised of a separate peptide library (data not shown). This demonstrates that the immunosignature can distinguish canine LSA patients from healthy dogs.

### The immunosignature predicts health status in an independent set of dogs

To test the predictive ability of the peptides identified above, additional LSA-B patients (n = 20) and healthy donors (n =22) were obtained. Serum from all LSA-B patients (n = 38) and healthy donors (n = 39) were randomized and used to probe the CIM10Kv2. Technical requirements necessitated that serum from all dogs be run on a second print run of the CIM10Kv2 and detected using a different secondary antibody due to product discontinuation by the original supplier. The 340 peptides selected above to separate LSA and healthy clearly separated the expanded test set of dogs, even though the print run and anti-IgG secondary antibody were different (Additional file 1: Figure S1). When the training set arrays from print run 1 were used to predict the test set from print run 2, the accuracy was 97%: one LSA-B patient was miscalled as healthy. This accuracy was the same whether the training set arrays were from the same or different batch than the test set.

To exclude the possibility that the distinction between LSA and healthy was an artifact of this division of training and test sets, the dogs were iteratively randomized into training (85%) and test (15%) sets. The training set was used to select peptides having a p value < 0.05 with FDR and a minimum fold change between classes of 10.0 fold. The peptides were then used to train an SVM and predict class membership of the test set. Over 10,000 randomizations into training and test sets, the median performance on the test set accuracy was 92 +/- 9.6%, sensitivity was 100 +/- 12.2% and specificity was 83 +/- 14.3%. The lower specificity was due to 3 healthy dogs that were consistently miscalled when included in the test set (Additional file 1: Table S1). To assess how much of the immunosignature is due to other factors, all healthy and LSA-B dogs were combined and divided based on gender and age. Separation of dogs into two classes based on age (division was 7 years old) yielded 14 significant peptides that were unable to classify the dogs on either a PCA or SVM. Further separation into male and female dogs yielded one significant peptide that was unable to classify in either a PCA or SVM. This suggests that the difference in immunosignature based on health or disease is due to the LSA and not other factors. Taken together, this demonstrates that the immunosignature is both capable of predicting an independent test set and is stable across array print runs and detection systems.

### The immunosignature can distinguish dogs with t cell lymphoma from those with b cell lymphoma

**Figure 2:**
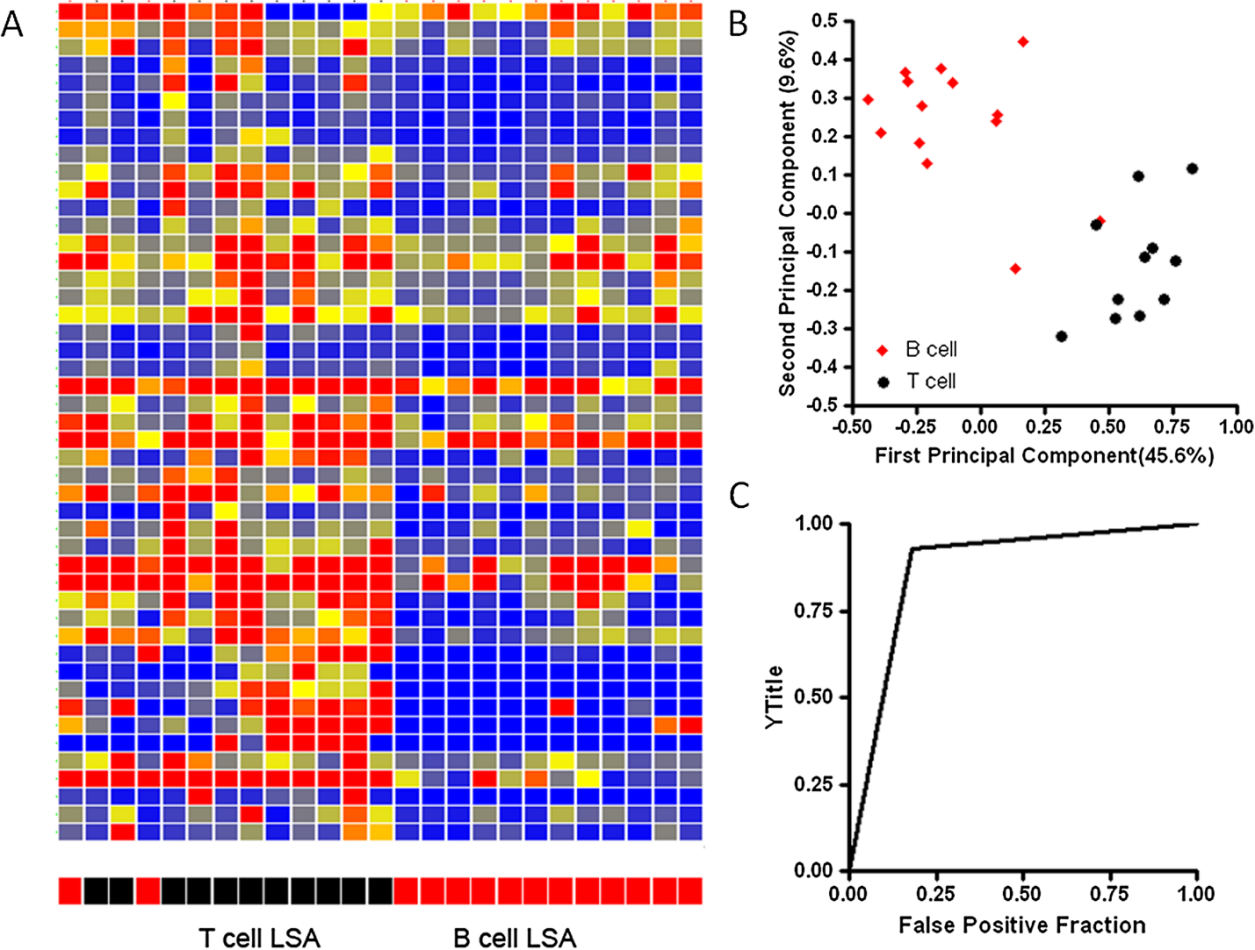
**The immunosignature distinguishes LSA-B from LSA-T.** A Student’s T-test (p < 0.05 with FDR) and a 1.5 fold change between classes were used to select 47 informative peptides. The distribution of intensities is shown in the Heatmap **(A)**. Colors represent the per peptide median normalized intensities. Yellow indicates the median, red five-fold above the median and blue 0.25 fold below the median. Each row represents a peptide and each column represents and individual. Individuals were clustered in GeneSpring using the Pearson correlation to each other. Variation among individuals based on the 47 peptides is shown in the PCA plot **(B)**, where the first two principal components are plotted. The classification efficacy is plotted in the ROC curve in **(C)**. Print run one and the KPL conjugate were used for this assay.

In dogs, LSA-T tends to be a more aggressive form of LSA than LSA-B [24], and determining this distinction can have impacts on both outcome and, in some cases, choice of treatment [25]. For this reason, immunophenotyping is commonly performed as part of initial staging in dogs with LSA. The immunosignature of the LSA-B (n = 14) and LSA-T (n =11 ) patients were compared using a Student’s T-test, and 47 peptides had a *p* value less than 0.05 with FDR and a minimum 1.5 fold difference between classes. Reactivity is shown in the heatmap in Figure 2A. Separation of LSA-B and LSA-T is shown in the PCA in Figure 2B. Leave one out cross validation was able to separate LSA and healthy with 88% accuracy: one member of each class was misidentified. A ROC curve is shown in Figure 2C. Interestingly, one of the serum samples initially identified as from a LSA-B patient clustered with the LSA-T patients and classified as a LSA-T patient. This patient was subsequently confirmed to have a CD3 positive LSA. A similar distinction was made using the CIM10Kv1 arrays (data not shown). This demonstrates that the immunosignature can distinguish LSA of B and T cell origin.

### Characterization of the individual lymphoma immunosignature

**Figure 3:**
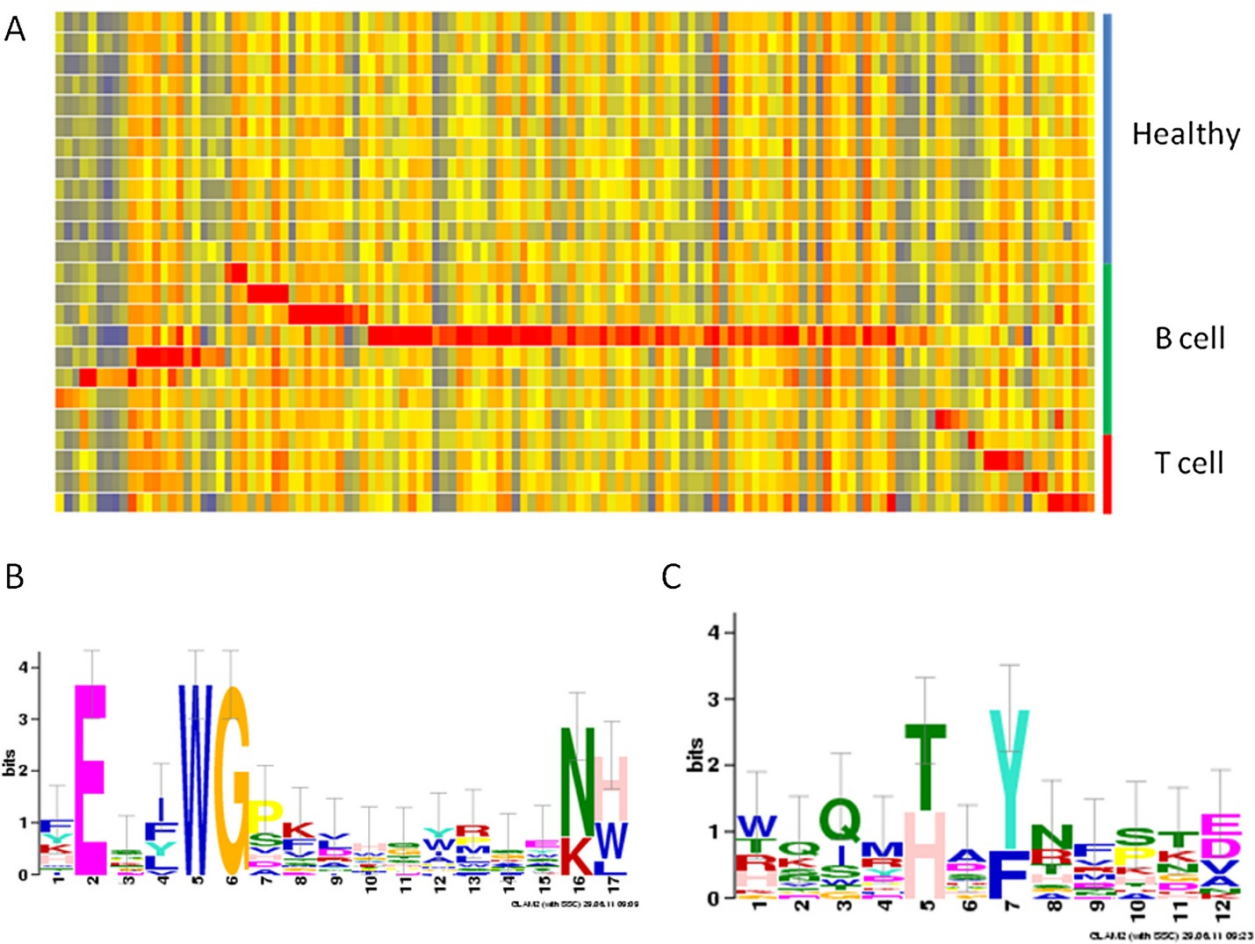
**Individual lymphoma signatures. (A)** Peptides comprising the unique elements of the immunosignatures of canine lymphoma patients are displayed in a false color heatmap. Red represents 5 fold greater than median, yellow represents the median and blue 5 fold below the median. Peptides ranked in the lowest quartile for coefficient of variation among healthy dogs were analyzed by correlation (greater than Pearson R of 0.9) to a recognition profile of 10 fold above the median in an individual and at the median for both healthy dogs and lymphoma patients. The GLAM2 pattern-matching algorithm was used to establish patterns for each dog. Representative patterns for LSA-B are shown in **(B & C)**. Print run one and the KPL conjugate were used for this assay.

Lymphoma is a clonal disease arising from the uncontrolled proliferation of a single lymphocyte [3]. We have observed individual immunosignatures in human myeloma, another clonal B cell disease (Stafford *et al.* in preparation). This raises the possibility of an immunosignature for each LSA patient in addition to the general class immunosignature. Such an immunosignature could be either from the antibody species produced by the B cell or the immune response to the surface markers or other cancer related antigens of the LSA cell. In the case of LSA-T there could be a unique antibody response to the T cell receptor of the LSA clone. Pattern matching analysis was done to identify peptides uniquely recognized by each dog.To reduce the influence of recent vaccines or infections, the peptides with the least variability in healthy donors (bottom quartile ranked on CV) were analyzed. The pattern used was the per peptide median for all dogs except the LSA patient being queried for which the value was set at 5 fold above the per peptide median. Peptides matching the profile with a Pearson correlation greater than 0.90 were defined as unique to that individual. A heatmap of the unique peptides is presented in Figure 3A. The median number of peptides identified in the LSA-B dogs was 8 (range: 3 to 71) and the median number of identified peptides in the LSA-T patients was 6 (range: 2 to 6). No peptides matching these profiles were bound in the healthy dogs. If these unique peptides are bound by a single antibody clone, then a motif could be present in the peptide list. Sequence motifs were identified using the GLAM2 algorithm and representative motifs are presented as logo plots in Figure 3B and C. Individual immunosignatures with associated motifs were also seen on the CIM10Kv1 (not shown). Taken together, these data agree with the clonal nature of the disease and suggest that the individual immunosignature may be the result of a single antibody clone, whether that produced by the involved B cell clone or to a unique antigen such as the BCR or TCR idiotype.

### Monitoring the immunosignature present at diagnosis marks remission but not relapse

To determine if the immunosignature could be used to monitor patients for early signs of relapse, we obtained sera from 12 dogs with LSA at the time of diagnosis, 3 months following treatment at which point they were clinically in remission, and at the time of relapse. The sera was run on the CIM10Kv2 and evaluated for changes both personal and subtype immunosignatures. Comparison of the individual immunosignatures between diagnosis and remission indicated that a median of 72 percent of the immunosignature decreased following initiation of treatment (Table 2). This suggests that the individual LSA immunosignature has utility in establishing or verifying remission. However, the antibody reactivity level of these peptides did not return to the level observed in normal dogs. This suggests that the immune response generated against the LSA may be maintained by the small number of cells that lead to the recurrence. Peptides decreasing at remission did not also increase at relapse. Survival of the minimal residual disease cells and the lack of the return of decreased peptides to pre-treatment levels suggest that the tumor could have been kept in check by the immune umbrella of the original tumor but new antigens and pathways enabled relapse.

**Table 2:** **Change in the individual immunosignature between diagnosis and reoccurrance** ^**1**^

				Portion of the signature decreasing by 3 months ^2^			Portion of signature returning to pretreatment Levels at relapse ^3^		
Individual	Type ^4^	DFI (days) ^5^	Peptides ^6^	Number ^7^	Percent ^8^	Median fold change at day 90	Number	Percent	Median fold change vs day 0
247690	B	174	3	0	0	1.21	0	0	0.78
248661	B	280	5	1	20	0.85	0	0	0.59
251332	B	273	10	7	70	0.59	0	0	0.42
251661	B	119	71	65	92	0.42	5	7	0.37
252343	B	257	11	9	82	0.46	1	9	0.49
253509	B	270	7	7	100	0.45	0	0	0.20
256744	B	112	3	1	33	0.87	1	33	0.94
257195	B	333	4	3	75	0.69	1	25	0.45
219623	T	185	2	1	50	0.78	1	50	0.95
238300	T	167	5	4	80	0.54	0	0	0.49
243388	T	270	3	3	100	0.37	3	100	1.42
251702	T	71	6	0	0	1.37	0	0	1.40

^1^The individual signature is defined as the number of peptides uniquely recognized by each individual compared to other dogs in the study.

^2^The number of peptides in the individual immunosignature that decreased in normalized RFI greater than the minimum detectable fold change of 0.76.

^3^The number of peptides with a normalized RFI that returned to within the minimum detectable fold change of 0.76 to 1.3x of the values at diagnosis by the time they were clinically out of remission.

^4^Phenotype of the lymphoma, B cell or T cell.

^5^Time between initiation of treatment and being clinically defined as out of remission.

^6^The number of peptides comprising the individual immunsignature.

^7^The number of peptides comprising the individual immunsignature that decreased at three months.

^8^Percent of the individual immunosignature that decreased at three months.

The subtype immunosignature was defined as the peptides increased in each phenotype. For the 177 peptides increased in LSA-B, a median of 25 +/- 14 peptides (14%) decreased at remission and 13 +/- 7 peptides increased from remission to relapse. Of the 173 peptides increased in LSA-T, a median of 35 +/- 20 peptides (20%) decreased upon remission and a median of 21.4 +/- 6 peptides (12%) increased between remission and relapse. Antibodies binding the subtype specific immunosignature were likely raised by a normal B cells against the LSA cell as part of the anti-LSA immune response. Clinical therapy either excises or chemically kills the LSA cell, removing the antigen that stimulated the normal B cell. Persistence of the immunosignature after removal of the LSA cell suggests that the normal B cell had differentiated to a long lived plasma cell and was unaffected by remission. Taken together, this indicates that the immunosignature can verify remission through the personalized signature, but other means are needed to detect recurrence.

### Further characterization of the B Cell lymphoma immunosignature

Retrospective surveys of LSA-T and LSA-B identified median DFIs of 2.5 months for LSA-T and 6.74 months for LSA-B following multi-agent chemotherapy [24]. In this study and in clinical practice, dogs with LSA-B tended to have a higher proportion of survivors at later months [24], indicating a need to further characterize the LSA-B immunosignature. The median raw feature intensity of arrays probed with serum from LSA-B patients and healthy donors were compared. The median raw feature intensity of the arrays probed with LSA-B were significantly lower (p = 1×10^-4^) than those probed with healthy donors. In prior studies we noted overall feature intensity increases in infection and as the concentration of antibodies applied to the array increases [15]. This suggests that either the overall amount of immunoglobulin or reactivity is depressed in LSA-B patients, fitting with clinical studies of NHL in humans, which report serum hypogammaglobemmia in 10 to 15% of patients [26, 27] and a 21% reduction of crude median IgG levels in DLBCL patients [27].

### The immunosignature of B Cell LSA at diagnosis is capable of predicting length of disease free interval in dogs entering remission

**Figure 4:**
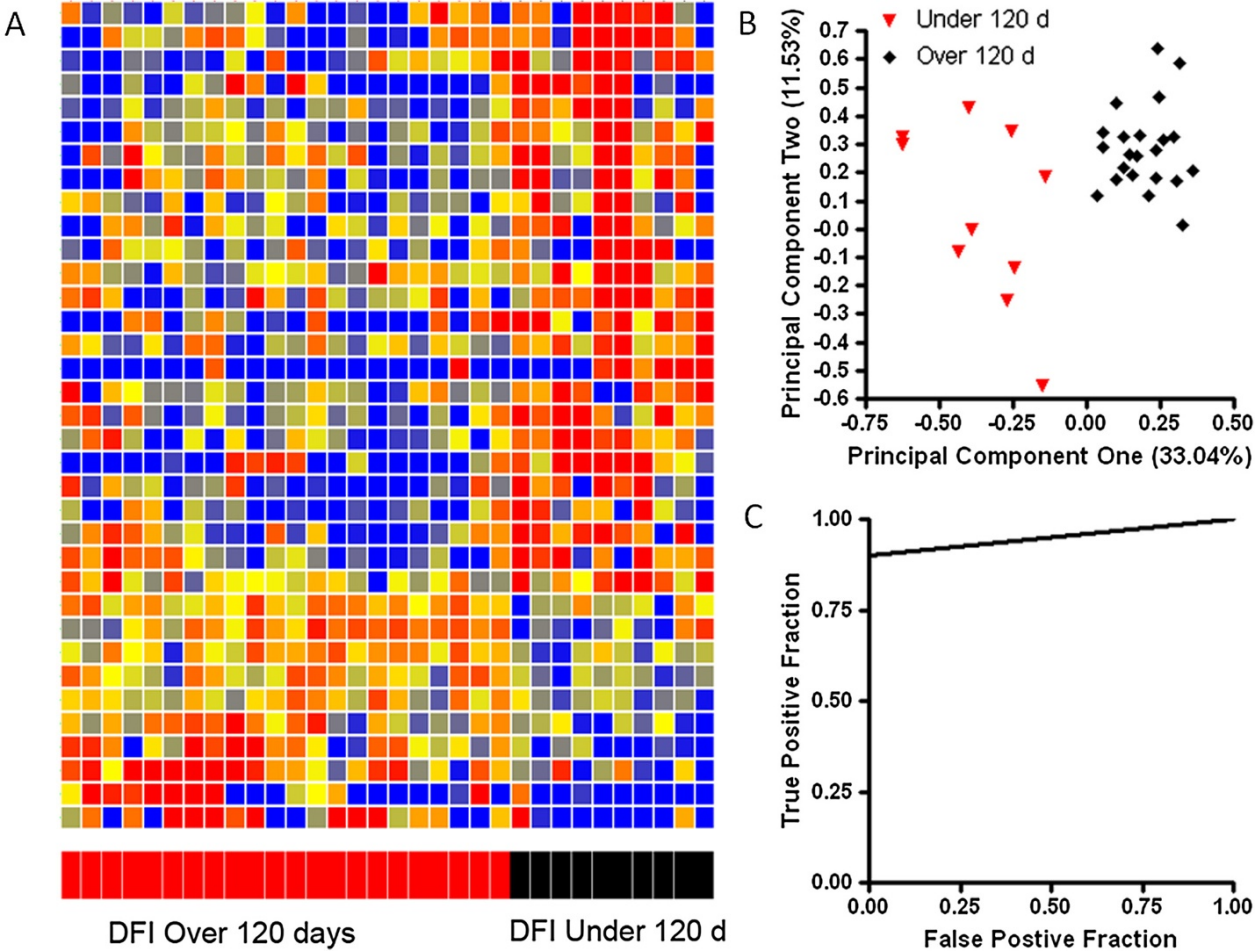
**The immunosignature at diagnosis can predict disease free interval.** A Student’s T-test (p < 0.05 with FDR) and a 1.5 fold change between classes was used to select 35 informative peptides. The distribution of intensities is shown in the Heatmap **(A)**. Colors represent the per peptide median normalized intensities. Yellow indicates the median, red two fold above the median and blue less than 0.8 fold below the median. Each row represents a peptide and each column represents and individual. Individuals were clustered in GeneSpring using distance measurement to each other. Variation among individuals based on the 35 peptides is shown in the PCA plot **(B)**, where the first two principal components are plotted. The classification efficacy is plotted in the ROC curve in **(C)**. Print run two and the Jackson conjugate were used for this assay.

Since changes in the individual or class immunosignatures following treatment were not indicative of relapse, we sought to determine if the immunosignature at diagnosis could predict time to relapse. The LSA-B patients were divided into those that relapsed in under 120 days (n = 10) and those that had a delayed relapse of over 120 days (n = 23). A Student’s T-test identified 35 peptides with a p value <0.05 with FDR between classes. A heatmap of the selected peptides is presented in Figure 4A. Separation of the patients based on DFI is presented in the PCA plot shown in Figure 4B. Note that the dogs having a longer DFI cluster more tightly than those with a short DFI, having median Mahalanobis distances to the class means of 13.66 and 17.27 respectively, indicating less variance in the under 120 day immunsignature than the over 120 day immunosignature. An SVM trained on the 35 peptides has a LOOCV accuracy of 97%. A ROC curve is presented in Figure 4C. This demonstrates that the immunosignature is capable of predicting the length of DFI at the time of diagnosis.

## Discussion

It has been well established that most cancers are capable of generating detectable cellular and humoral immune responses, although they are insufficient to control the disease. This observation has led to the exploration of immune-based cancer therapies in humans and dogs, including in canine LSA [28–31]. In the present study we have used immunosignatures to characterize canine LSA and evaluated the immunosignature technology as a diagnostic. We have demonstrated that canine LSA patients can be readily distinguished from healthy dogs. The immunosignature of LSA was capable of predicting an independent test set with high accuracy and was robust to print run and detection system changes. Furthermore, the distinction between B and T-cell LSA was able to be determined from the immunosignature. Individualized immunosignatures were also observed in the LSA patients at diagnosis, which declined as patients entered remission. The individualized immunosignature did not return at relapse for all dogs; however, the immunosignature at diagnosis was capable of predicting the length of DFI. Taken together, this study demonstrates the clinical utility of the immunosignature as a multilevel diagnostic for LSA.

Lymphoma is a clonal disease arising from an abnormally proliferating B or T cell. Despite the clonal origin of the disease, a LSA specific immunosignature was identifiable. In humans, certain variable region genes are prevalent in LSA [32–34] and superantigens are LSA associated [35], raising the possibility that the immunosignature is that of a causal immunological insult. Proteomic studies in humans have identified distinct protein expression profiles that are informative not only for LSA, but clinical subtype [36, 37]. This raises the possibility that the immunosignature is reflecting cancer antigens associated with LSA. In individual dogs, an individual immunosignature was additionally detected and may be the product of the individual B cell clone while the class immunosignature was reflective of the underlying cancer biology. The influence of the antibody produced by the individual B cell clone is reflected in the immunosignature distinguishing LSA-B and LSA-T, where the LSA-T patients cluster together much more tightly on the PCA than do LSA-B patients. It is of note that the individual signature may indicate the number of peptides bound per antibody.

We observed that the personalized immunosignature declined as the dog entered remission along with the common LSA-B signature fits with what is known of this cancer. The causal B cell clone that forms the LSA is present in abundance at diagnosis and is excised or chemically reduced to barely detectible levels at remission, yet antibodies against the LSA persist. Serum IgG in dogs has a half-life of 8 to 12 days [38], indicating that at the three-month sampling, serum levels of the anti-LSA antibody species could have reduced to 1 to 6% of the diagnosis titer. This suggests that the anti-LSA antibody response may have differentiated to long lived plasma cells which have a 140 day half-life [39] and are largely unaffected by immunosuppressive chemotherapy [40, 41]. That the personalized immunosignature did not increase upon relapse suggests that either other antigens are involved in triggering relapse of a dormant tumor cell, somatic hypermutation has occurred, or the relapse is actually a second clonal lineage initiating an antigenically distinct LSA tumor. The disease free interval in both dogs and humans is a period of watchful waiting, punctuated with frequent recheck examinations, blood draws and imaging. The immunosignature at diagnosis is capable of identifying dogs having an aggressive LSA that relapsed in less than four months from those with a less aggressive form.

Conversion of a microarray-based assay to a diagnostic is reliant on the robustness of the platform to differences in technician, print batch and detection system. In our training and test set, we evaluated the sum effect of these conditions. The training set was initially run as one batch then repeated in the second batch with the test set. When the SVM was trained using either training set batch, the test set was predicted with >94% accuracy. This demonstrates that as a practical diagnostic assay, a single patient could be normalized to a co-run standard and the immunosignature compared to a database, thereby alleviating the impractical possibility of having to run a 60 sample training set with each assay. Further enabling the transition to a clinical diagnostic is the ability of antibodies eluted from dried blood spots to perform in the immunosignature assay [17].

There are multiple other techniques that have been established for the determination of immunophenotype in canine LSA. These include immunohistochemistry, immunocytochemistry, flow cytometry, and PCR for antigen receptor rearrangement (PARR) [42]. While all are associated with acceptable sensitivity and specificity, all require tumor samples and thus would not be suitable as a screening or early detection test. Real-time PCR of peripheral blood, using reagents specific for a patient’s particular antigen receptor gene rearrangement, has also been used for remission status monitoring and relapse detection in canine LSA [43, 44]; however, this is labor and time intensive and requires the generation of custom tools for each patient, and would also not be suitable as a screening test.

## Conclusions

This study illustrates the immunosignature as an improvement over current veterinary serodiagnostics for LSA. As opposed to tests which either non-specifically indicate a cancer by measuring thymidine kinase activity [45] or rely on biomarkers [46], the immunosignature is information rich. From a single assay, the patient could potentially be diagnosed as having LSA or not, whether the LSA is of B or T cell lineage and whether the DFI following chemotherapy will be short or long. Each of these points of information is critical for the treating veterinarian. A negative immunosignature for LSA aids in the differential diagnosis, B cell LSA and T cell LSA may be treated differently, and knowing at diagnosis that the DFI will be short may provide important prognostic information to the pet owner and veterinarian. Given the similarity between both disease and immune system function in dogs and humans, the immunosignature is expected to provide the same information to physicians and their patients.

### Statement of translational relevance

This paper demonstrates that the serum antibody repertoire produces a unique binding pattern or immunosignature for lymphoma on a random peptide array. The immunosignature is descriptive for B or T cell lymphoma and estimating duration of remission following treatment. Thus the immunosignature has the capability to provide multiple levels of prognostic information to the patient and clinician.

## Electronic supplementary material

Additional file 1: **The immunosignature of canine lymphoma: characterization and diagnostic application supplemental material.** (DOCX 243 KB)

## Abbreviations

NHL: Non-Hodgkin lymphoma
LSA: B or T cell lymphoma
LSA-B: B cell lymphoma
LSA-T: T cell lymphoma
DFI: Disease free interval
SVM: Support vector machine
LOOCV: Leave one out cross validation
FDR: Benjamani and Hochberg False Discovery Rate correction.
