# Supplementary material for: The immunosignature of canine lymphoma: characterization and diagnostic application
Source: BMC Cancer. 2014 Sep 8;14:657. doi: 10.1186/1471-2407-14-657 (PMC4168252; doi:10.1186/1471-2407-14-657)
Supplement: Supplementary file 1 — Additional file 1: The immunosignature of canine lymphoma: characterization and diagnostic application supplemental material. (DOCX 243 KB) [file 12885_2014_4845_MOESM1_ESM.docx]

The Immunosignature of Canine Lymphoma: Characterization and Diagnostic Application

Supplemental Material

Table S1. Individual results of dogs predicted as healthy or B cell lymphoma in the iterative training and testing.^1^

| **Healthy** | | | |  | **B Cell Lymphoma** | | | |
| --- | --- | --- | --- | --- | --- | --- | --- | --- |
| **Individual** | **Correct^2^** | **Incorrect^3^** | **Accuracy^4^** |  | **Individual** | **Correct** | **Wrong** | **Accuracy** |
| *18* | 1511 | 120 | 92.64 |  | *1090* | 1362 | 194 | 87.53 |
| *31* | 1212 | 383 | 75.99 |  | *1093* | 1539 | 0 | 100.00 |
| *33* | 1593 | 1 | 99.94 |  | *1137* | 336 | 1201 | 21.86 |
| *35* | 471 | 1146 | 29.13 |  | *1144* | 1523 | 19 | 98.77 |
| *36* | 1526 | 151 | 91.00 |  | *1165* | 1532 | 14 | 99.09 |
| *4* | 31 | 1575 | 1.93 |  | *1167* | 1500 | 0 | 100.00 |
| *42* | 1575 | 0 | 100.00 |  | *13* | 1543 | 0 | 100.00 |
| *46* | 22 | 1591 | 1.36 |  | *231546* | 1586 | 0 | 100.00 |
| *49* | 1363 | 232 | 85.45 |  | *246620* | 1569 | 5 | 99.68 |
| *6* | 1484 | 95 | 93.98 |  | *247645* | 1193 | 310 | 79.37 |
| *201* | 1622 | 0 | 100.00 |  | *247659* | 1372 | 111 | 92.52 |
| *202* | 1590 | 27 | 98.33 |  | *247690* | 1470 | 1 | 99.93 |
| *203* | 1593 | 0 | 100.00 |  | *248661* | 1462 | 0 | 100.00 |
| *204* | 786 | 865 | 47.61 |  | *250947* | 1434 | 15 | 98.96 |
| *205* | 1587 | 1 | 99.94 |  | *251181* | 1575 | 0 | 100.00 |
| *206* | 1449 | 150 | 90.62 |  | *251332* | 1376 | 141 | 90.71 |
| *207* | 21 | 1602 | 1.29 |  | *251661* | 561 | 993 | 36.10 |
| *208* | 1613 | 1 | 99.94 |  | *252343* | 1280 | 220 | 85.33 |
| *209* | 1516 | 30 | 98.06 |  | *252353* | 1567 | 0 | 100.00 |
| *210* | 1605 | 2 | 99.88 |  | *252590* | 1336 | 244 | 84.56 |
| *211* | 1558 | 0 | 100.00 |  | *253357* | 1365 | 236 | 85.26 |
| *212* | 1424 | 207 | 87.31 |  | *253509* | 1431 | 172 | 89.27 |
| *213* | 1643 | 29 | 98.27 |  | *255800* | 1399 | 108 | 92.83 |
| *214* | 928 | 759 | 55.01 |  | *256744* | 1501 | 4 | 99.73 |
| *215* | 1676 | 0 | 100.00 |  | *256807* | 1050 | 490 | 68.18 |
| *216* | 1594 | 20 | 98.76 |  | *257195* | 1536 | 34 | 97.83 |
| *217* | 1682 | 22 | 98.71 |  | *258923* | 647 | 945 | 40.64 |
| *218* | 1502 | 174 | 89.62 |  | *260583* | 1058 | 514 | 67.30 |
| *219* | 1577 | 0 | 100.00 |  | *262527* | 1542 | 16 | 98.97 |
| *220* | 1652 | 0 | 100.00 |  | *262725* | 1596 | 0 | 100.00 |
| *221* | 1671 | 0 | 100.00 |  | *262948* | 1525 | 0 | 100.00 |
| *222* | 1626 | 0 | 100.00 |  | *263141* | 1518 | 30 | 98.06 |
| *223* | 1616 | 0 | 100.00 |  | *263293* | 1505 | 0 | 100.00 |
| *224* | 1644 | 0 | 100.00 |  | *263451* | 1536 | 0 | 100.00 |
| *225* | 1643 | 0 | 100.00 |  | *263814* | 1484 | 63 | 95.93 |
| *226* | 1593 | 13 | 99.19 |  | *671* | 1524 | 3 | 99.80 |
| *227* | 1605 | 0 | 100.00 |  | *819* | 1527 | 11 | 99.28 |
|  |  |  |  |  | *822* | 1450 | 71 | 95.33 |
|  |  |  |  |  | *863* | 1352 | 173 | 88.66 |

^1^Dogs were iteratively divided into two sets (15% training and 85%) for 10,000 iterations. The training set was used to select peptides and train an SVM for prediction of the test set.

^2^ The number of times a dog was predicted correctly when in the training set

^3^ The number of times a dog was predicted incorrectly when in the training set

^4^ The individual accuracy for a given dog

**
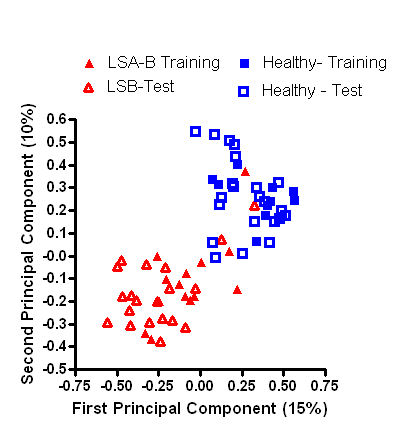
**

**Figure S1. Classification of an independent test set into LSA patients and healthy dogs.** The 340 peptides identified as informative between LSA and healthy dogs (p<0.05 with FDR and greater than 1.5 fold difference between classes) were used to train a support vector machine and predict a test set of dogs. The variance of all dogs in both the training set (solid symbols) and test set (open symbols) are presented in a principal component analysis. The first two principal components are presented as the x and y scalar values and each symbol represents the replicate average for a single dog. Peptides were selected on the training set using the KPL conjugate. The Jackson conjugate was used for both the training and test set in this assay.
